# ATR-FTIR spectroscopy in blood plasma combined with multivariate analysis to detect HIV infection in pregnant women

**DOI:** 10.1038/s41598-020-77378-3

**Published:** 2020-11-19

**Authors:** Lidiane G. Silva, Ana F. S. Péres, Daniel L. D. Freitas, Camilo L. M. Morais, Francis L. Martin, Janaina C. O. Crispim, Kassio M. G. Lima

**Affiliations:** 1 Institute of Chemistry, Biological Chemistry and Chemometrics, Federal University of Rio Grande Do Norte, Natal, RN 5072-970 Brazil; 2 Department of Clinical and Toxicological Analysis, Federal University of Rio Grande Do Norte, Natal, RN 59072-970 Brazil; 3 Maternity School Januário Cicco, Federal University of Rio Grande Do Norte, Natal, RN 59072-970 Brazil; 4 School of Pharmacy and Biomedical Sciences, University of Central Lancashire, Preston, PR1 2HE UK; 5 Biocel Ltd, Hull, HU10 7TS UK

**Keywords:** Biological techniques, Optical spectroscopy, Infrared spectroscopy, Translational research

## Abstract

The primary concern for HIV-infected pregnant women is the vertical transmission that can occur during pregnancy, in the intrauterine period, during labour or even breastfeeding. The risk of vertical transmission can be reduced by early diagnosis. Therefore, it is necessary to develop new methods to detect this virus in a quick and low-cost fashion, as colorimetric assays for HIV detection tend to be laborious and costly. Herein, attenuated total reflection Fourier-transform infrared (ATR-FTIR) spectroscopy combined with multivariate analysis was employed to distinguish HIV-infected patients from healthy uninfected controls in a total of 120 blood plasma samples. The best sensitivity (83%) and specificity (92%) values were obtained using the genetic algorithm with linear discriminant analysis (GA-LDA). These good classification results in addition to the potential for high analytical frequency, the low cost and reagent-free nature of this method demonstrate its potential as an alternative tool for HIV screening during pregnancy.

## Introduction

The human immunodeficiency virus (HIV) is the cause of a sexually transmitted infection that compromises the immunological system. This virus, belonging to the *Lentiviridae* family, attacks the cells of the immunological system, especially the T lymphocyte CD4 + cells, making them vulnerable to other infections^1^. The decrease of T lymphocytes CD4 + cells by HIV is one of the main causes of AIDS (acquired immune deficiency syndrome)^2^.

HIV was first identified in the 1980′s and since then it has affected millions of people, becoming a major public health problem^3^. Worldwide, around 38 million people are infected with HIV, from which 1.8 million are children and 35 million are adults^4^. According to the epidemiological bulletin from the Brazilian Ministry of Health^5^, 116,292 cases of gestational HIV were diagnosed in Brazil between 2000 and 2018, from which 7,888 cases were recorded in 2017.

One of the main concerns for HIV-infected pregnant women is the viral transmission from the mother to the child^3^. This is termed vertical transmission and can occur during pregnancy (intrauterine), during labour or during breastfeeding. The risk of vertical transmission can be reduced by early diagnosis whereupon antiretroviral treatment can be implemented to reduce viral load. Early HIV diagnosis has increased 21% in the last 10 years, enabling adequate treatment for women during pregnancy, thus improving their quality of life and reducing the risk of vertical transmission. Between 2007 and 2017, prenatal diagnosis resulted in an increase from 2.3 cases per thousand live births to 2.8 cases per thousand live births^5^.

The diagnosis of gestational HIV can be made in blood using immunoassays such as immunochromatography (a rapid test) or the ELISA (enzyme-linked immunosorbent assay) test, which enables one to score both antibodies and antigens. The viral load in HIV-infected patients are estimated > 1,000–1,500 copies/mL^6,7^. Colorimetric assays to detect HIV have a high-cost and require labour-intensive procedures, since each metabolite is detected one at a time^8^.

Spectrometric techniques have been used as metabolomic tools to discriminate biofluids derived from infected *vs.* healthy patients. Mass spectrometry (MS) and nuclear magnetic resonance (NMR) are the most widely used techniques. Although having high sensitivity and specificity, these techniques have a high-cost and detailed sample preparation is often required^2^. Attenuated total reflection Fourier-transform infrared (ATR-FITR) spectroscopy is a fast, low-cost, non-destructive and reagent-free alternative to these spectrometric techniques, being able to generate a fingerprint spectrum containing both structural and concentration information for the constituent metabolites in the sample^9^.

There remain a limited number of applications using ATR-FTIR spectroscopy to detect HIV-infected samples. Sitole et al.^8^ showed for the first time metabolic differences between healthy uninfected and infected groups using ATR-FTIR spectroscopy where chemometric tools were employed to distinguish healthy uninfected *vs.* HIV-infected patients, in addition to distinguishing infected patients with or without antiretroviral treatment. Herein, ATR-FTIR spectroscopy combined with multivariate analysis is used to discriminate healthy uninfected and HIV-infected pregnant women based on their blood plasma spectra. Different chemometric techniques were tested, including approaches based on principal component analysis (PCA), successive projections algorithm (SPA) and genetic algorithm (GA).

## Results

**Figure 1:**
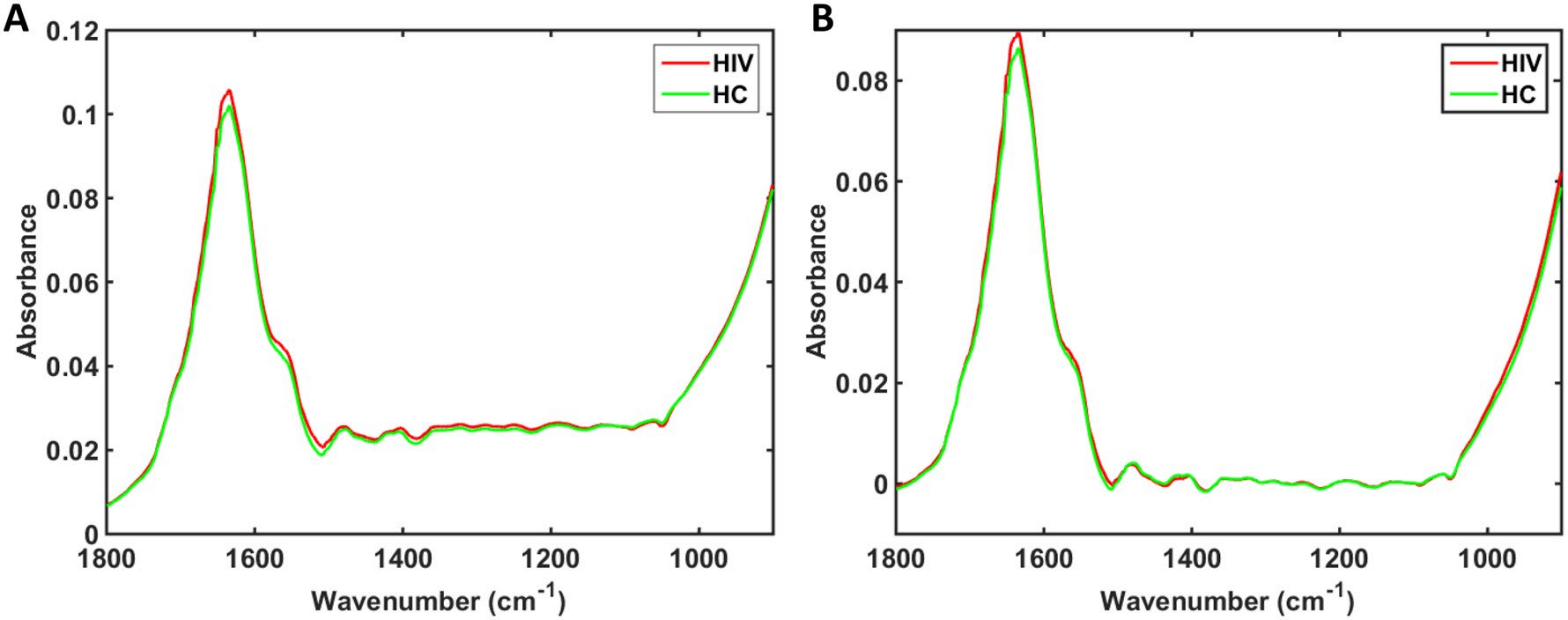
Infrared (IR) spectra. (**A**) Mean raw IR spectra in the bio-fingerprint region (1800–900 cm^−1^) for HIV-infected (HIV) and healthy uninfected controls (HC) samples. (**B**) Mean pre-processed IR spectra (AWLS baseline correction) in the bio-fingerprint region (1800–900 cm^−1^) for HIV-infected (HIV) and healthy uninfected controls (HC) samples.

The infrared (IR) spectra of blood plasma for control (healthy uninfected pregnant women) and HIV-infected pregnant women are shown in Fig. 1A. A total of 120 samples were analysed, generating 360 spectra (3 spectra per sample). The 3 spectra per sample were averaged before model construction. The sample set was composed of 80 healthy uninfected controls and 40 HIV-infected samples. Study participant information and demographics is shown in Table 1.

**Table 1:** Study participant information and demographics.

	Group	
	Case (HIV-infection)	Control uninfected
Age (mean ± SD)	28 ± 6	31 ± 6
Gestational age (weeks ± SD)	21 ± 7	24 ± 7
Smoking, *n* (%)		
Yes	4 (10.3%)	0
No	35 (89.7%)	35 (100%)
Drugs use, *n* (%)		
Yes	7 (18%)	3 (8.57%)
No	32 (82%)	33 (94.3%)

In the region between 1800 to 900 cm^−1^ (bio-fingerprint region), the spectra show characteristic absorptions: Amide I band at 1635 cm^−1^ ^10^, an arm at 1560 cm^−1^ (C = O, Amide II)^11^ and three small intensity absorptions at 1480 cm^−1^ (C-H asymmetric deformation of methyl groups), 1404 cm^−1^ (COO − symmetric stretching of proteins and lipids) and 1060 cm^−1^ (C-O nucleic acids)^10^. To correct for baseline distortions, the raw spectral data were pre-processed by automatic weighted least squares (AWLS) baseline correction (Fig. 1B). Due to the high similarity between the spectral features for both groups (control uninfected and HIV infected), chemometric approaches were employed to identify spectral features responsible for class differentiation.

PCA with linear discriminant analysis (PCA-LDA), quadratic discriminant analysis (PCA-QDA) or support vector machines (PCA-SVM); successive projections algorithm with linear discriminant analysis (SPA-LDA), quadratic discriminant analysis (SPA-QDA) or support vector machines (SPA-SVM); and, genetic algorithm with linear discriminant analysis (GA-LDA), quadratic discriminant analysis (GA-QDA) or support vector machines (GA-SVM) were tested as feature extraction/selection and classification tools.

The performance for each algorithm tested was evaluated by calculating the accuracy, sensitivity, specificity, F-score and G-score for the test set. Both classes were separated into training (70%) and test (30%) sets using the Kennard-Stone algorithm^12^, whereby the training set was used for model construction and the test set for model validation. The classification metrics in the test set for each algorithm is shown in Table 2.

**Table 2:** Quality parameters calculated in the test set to classify healthy uninfected controls *vs.* HIV-infected samples.

Model	AC	SENS	SPEC	F-Score	G-Score
PCA-LDA	70	67	71	69	69
PCA-QDA	58	83	46	59	62
PCA-SVM	81	83	79	81	81
SPA-LDA	75	50	88	64	66
SPA-QDA	64	8	92	15	28
SPA-SVM	67	8	96	15	87
GA-LDA	89	83	92	87	87
GA-QDA	64	8	100	15	29
GA-SVM	66.7	0	100	0	0

AC = accuracy, SENS = sensitivity, SPEC = specificity.

From all models tested, the best classification performances were obtained by PCA-SVM, SPA-LDA and GA-LDA. PCA-SVM showed a good accuracy (81%) and sensitivity (83%), but the specificity was < 80% (79%) in the test. SPA-LDA selected 22 spectral wavenumbers for class differentiation, generating a good specificity (88%), but poor accuracy (75%) and sensitivity (50%). GA-LDA was the best classification algorithm, with an accuracy of 89%, and sensitivity and specificity of 83% and 92%, respectively. The F-score (87%) and G-score (87%) show consistency, indicating that the classification performance was not affected by imbalanced class sizes.

**Figure 2:**
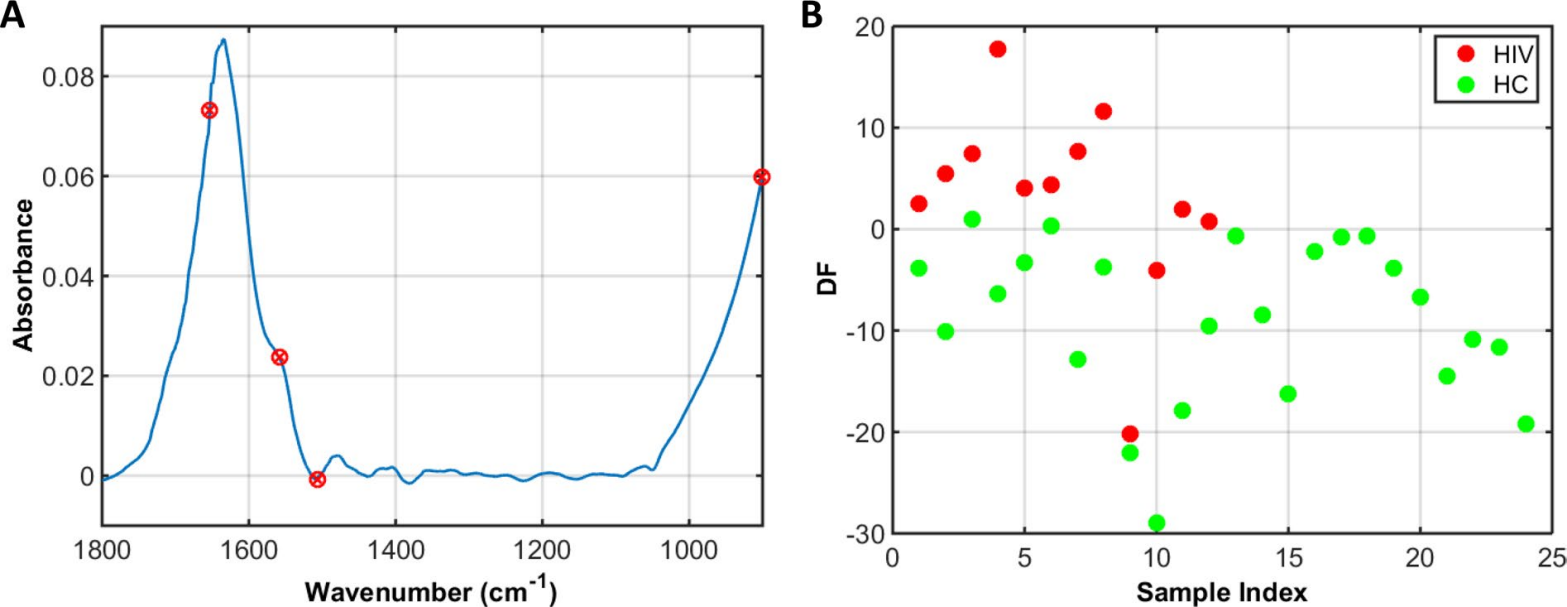
GA-LDA results. (**A**) Selected wavenumbers (901, 1506, 1558, 1653 cm^−1^) responsible for class separation. (**B**) Discriminant function (DF) for the samples in the test set, where HIV stands for HIV-infected samples and HC for healthy uninfected controls.

GA-LDA selected 4 spectral wavenumbers, among the 467 in the entire bio-fingerprint spectral range, for class differentiation. These wavenumbers were: 1653, 1558, 1506 and 901 cm^−1^ (Fig. 2A). The GA-LDA discriminant function (DF) in the test set is depicted in Fig. 2B, while the tentative biomarkers assignment^9^ for the 4 selected wavenumbers are shown in Table 3.

**Table 3:** Wavenumbers selected by GA-LDA to discriminate healthy uninfected controls *vs.* HIV-infected samples.

Selected wavenumber (cm^−1^)	Tentative assignment
901	Phosphodiester stretching
1506	Ring base
1558	Amide II (N–H bending vibration)
1653	Amide I (C = O stretching)

Tentative assignment based on Movasaghi et al.^10^.

## Discussion

HIV infection remains a major cause of morbidity and mortality worldwide^13^, being mainly transmitted during unprotected sexual intercourse, as well as by the sharing of infected needles or syringes, blood transfusion, and by mother-to-child transmission during pregnancy, labour or breastfeeding^14^. This latter phenomenon is termed vertical transmission and is the focus of this study.

The vertical transmission rate ranges from 15 to 45% worldwide, although this figure can be reduced to < 5% if effective intervention measures are applied. Early diagnosis and correct treatment with anti-retrovirals are among these measures^15^. In Brazil, a considerable proportion of HIV diagnosis for the female population occurs during pregnancy, where diagnosis via an anti-HIV serological screening is performed^15^. This reflects the adequacy of the health policy in prenatal care, although conscientisation campaigns on measures to prevent sexually transmitted infections and unintended pregnancy must still be disseminated widely to improve population health^16^.

Early diagnosis is of fundamental importance towards maintaining both the mother’s and new-born’s health with the intention of reducing vertical transmission risks. Diagnoses are commonly performed by screening using immunoassays; that is, tests that seek for the presence of anti-HIV antibodies and/or antigen p24 (protein in the virus nucleocapsid). These tests normally have high sensitivity and specificity, and are available in rapid-test formats (*e.g.*, immunochromatography), where the result is obtained within 30 min, or in the ELISA format^17^. Despite these advantages, current biochemical tests require skilled technicians, the use of unstable reagents highly dependent on storage conditions, and production of biochemical waste. Using these approaches, there is a possibility of HIV detection after 2 to 3 weeks from initial viral exposure.

There are other more complex tests generally used as complementary and/or confirmatory tests when screening tests diverge, such as the Western Blot, Imunoblot, Fast Imunoblot and molecular biology testing^18^, although these tests have a substantially higher cost and require highly-skilled labour and specialist facilities. Molecular biology testing is of fundamental importance for early diagnosis, since it is capable of identifying the virus’ presence before the period of production of immunoglobulins through the amplification of viral nucleic acids.

ATR-FTIR spectroscopy is capable of detecting metabolic changes after viral infection^19^, and has been successfully used to identify a range of viruses, such as Zika, Dengue and Chikungunya^20,21^. Herein, ATR-FTIR spectroscopy harnessed to chemometric techniques was employed to detect HIV infection in pregnant women. The spectral information responsible for class differentiation is probably associated with metabolic changes caused by the viral infection.

Since the samples were measured in liquid state, water is a possible interference that may compress and mask some of the absorption bands, altering their profiles in comparison with dessicated samples. Water mainly affects the high-region of the IR spectrum, with strong bands at 3,300–3,400 cm^−1^ (hydrogen-bonded O–H stretching) and 3,600–3,650 cm^−1^ (free O–H stretching)^22^. Since, we worked with the bio-fingerprint region (900–1,800 cm^−1^) only, these main water bands were removed. However, water has an absorbance band within the bio-fingerprint region at 1646 cm^−1^, which broadens the Amide I band and creates a shoulder^23^. In addition, absorbances < 1,000 cm^−1^ tend to greatly increase in a liquid environment, hence, compressing the bands between 1,200–1,500 cm^−1^ ^23^.

The virus identification was successfully performed on 40 HIV-infected samples *vs.* 80 healthy controls using GA-LDA algorithm, with a classification accuracy of 89%. For details, see Supporting information. Four spectral wavenumbers were responsible for class separation based on GA-LDA (Table 2): these included phosphodiester stretching bands at 901 cm^−1^, 1506 cm^−1^ (Ring base), protein bands at 1653 cm^−1^ (Amide I) and 1558 cm^−1^ (Amide II). The sample discrimination caused by changes in these protein features can be justified by the increase of immunoglobulin levels to fight the infection, since when viral infection happens, the immunological system is hyper-activated, hence, increasing production of immunoglobulin^13^.

Sitole et al.^8^ using ATR-FTIR spectroscopy also assigned the observed spectral changes between uninfected controls and HIV-infected patients to spectral regions associated with lipids, carbohydrates and proteins. Some protein wavenumbers responsible for class discrimination were: 1652 and 1650 cm^−1^ (Amide I) and 1500 and 1504 cm^−1^ (Amide II).

Herein, ATR-FTIR spectroscopy with multivariate analysis was able to accurately predict HIV-infected pregnant women based on blood plasma, showing the potential of this method for early detection of HIV in a fast and reagent-free approach. Successful development of this method in a clinical environment could aid early diagnosis of gestational HIV and help treatment.

## Methods

### Samples

This study was approved by the ethics committee at the Maternidade Escola Januário Cicco (MEJC) from the Federal University of Rio Grande do Norte with the protocol number 1.808.891 and informed consent was obtained from all subjects. Also, all the methods carried out in this study were by the approved guidelines. The patients were selected according to the automatic request that they underwent prenatal appointments, where the patients were asked whether they were willing to participate in this study. If accepted, an Informed Consent Form was read and signed by the participants. The participants answered a questionnaire and some clinical details were obtained from their medical records after consent. The inclusion criteria were: pregnant women between 12 to 30 weeks of pregnancy (Table 1), with or without HIV diagnosed and undergoing prenatal care.

Forty blood plasma samples (*n* = 40) were collected from HIV-positive patients (HIV-infected group) and eighty blood plasma samples (*n* = 80) from healthy uninfected controls. The samples were collected from May 2017 to March 2019 from patients attending the Maternidade Escola Januário Cicco (MEJC) and the Centro Reprodutivo Dra. Leide Morais in Natal/Brazil. Blood samples were collected into EDTA tubes and, thereafter, centrifuged for 10 min at 1500 rpm at room temperature to separate the blood plasma.

### Spectral acquisition

An FTIR IR-Affinity-1S spectrometer (Shimadzu Corporation, Japan) was used for spectral acquisition attached to an ATR accessory containing a diamond crystal as reflector element. The instrument was setup to run 32 scans per sample with a spectral resolution of 4 cm^−1^. Measurements were carried out in triplicate.

The IR spectra were recorded between 4000 to 600 cm^−1^ using 10 µL of sample directly applied onto the ATR crystal. Before starting the measurements, the ATR crystal was cleaned with ethanol 70% v/v and acetone; and before each new sample, the ATR crystal was cleaned with ethanol 70% v/v. A background spectrum was taken before measuring every new sample to account for environmental changes.

### Data analysis

The spectral data analysis was performed within MATLAB R2014b environment (MathWorks, Inc., USA) using the PLS-Toolbox version 7.9.3 and lab-made routines. The data were pre-processed by automatic weighted least squares (AWLS) baseline correction and organised so that the samples were split into training (70%) and test (30%) sets using the Kennard-Stone uniform sampling algorithm^12^. The following feature extraction/selection and classification algorithms were tested to discriminate healthy controls *vs.* HIV-infected samples: principal component analysis with linear discriminant analysis (PCA-LDA), quadratic discriminant analysis (PCA-QDA) and support vector machines (PCA-SVM); successive projections algorithm with linear discriminant analysis (SPA-LDA), quadratic discriminant analysis (SPA-QDA) and support vector machines (SPA-SVM); and genetic algorithm with linear discriminant analysis (GA-LDA), quadratic discriminant analysis (GA-QDA) and support vector machines (GA-SVM).

In PCA-LDA/QDA/SVM, the principal component analysis (PCA) scores^24^ are used as input variables for linear discriminant analysis (LDA), quadratic discriminant analysis (QDA) or support vector machines (SVM)^25,26^. The successive projections algorithm (SPA)^27^ and the genetic algorithm (GA)^28^ are feature selection algorithms, where the first works by selecting spectral wavenumbers that minimises the data collinearity, and the latter selecting spectral wavenumbers according to an evolution-based process, where sets of wavenumbers undergo combinations, crossover and mutations until a fitting criterion is reached, which is the minimisation of the following cost-function $G$ ^29^:

1 $$G = \frac{1}{{N_{V} }}\mathop \sum \limits_{n = 1}^{{N_{V} }} g_{n}$$

where $N_{V}$ is the number of validation samples, and $g_{n}$ is defined as:

2 $$g_{n} = \frac{{r^{2} \left( {x_{n} , m_{I\left( n \right)} } \right)}}{{dmin_{I\left( m \right) \ne I\left( n \right)} r^{2} \left( {X_{n} ,m_{I\left( m \right)} } \right)}}$$

where the numerator is the squared Mahalanobis distance between object $x_{n}$ of class index $I\left( n \right)$ and the sample mean $m_{I\left( n \right)}$ of its true class; and the denominator is the squared Mahalanobis distance between object $x_{n}$ and the center of the closest wrong class. GA was performed using 100 generations with 200 chromosomes each, with a mutation and crossover probability of 10% and 60%, respectively. LDA, QDA and SVM were used as classification techniques coupled SPA and GA selected variables.

LDA and QDA are discriminant analysis algorithms based on a Mahalanobis distance calculation between the samples. The main difference between them is that LDA assumes classes having similar variance structures, by using a pooled covariance matrix, while QDA assumes classes having different variance structures by using the variance–covariance matrix of each class individually for calculation^25^. The LDA classification score for sample $i$ of class $k$ ($L_{ik}$) is calculated in a non-Bayesian as follow^25,30^:

3 $$L_{ik} = \left( {{\mathbf{x}}_{i} - { }{\overline{\mathbf{x}}}_{k} } \right)^{{\text{T}}} {\mathbf{C}}_{{{\text{pooled}}}}^{ - 1} \left( {{\mathbf{x}}_{i} - { }{\overline{\mathbf{x}}}_{k} } \right)$$

where ${\mathbf{x}}_{i}$ is a vector with the input variables for sample $i$; ${\overline{\mathbf{x}}}_{k}$ is the mean of class $k$; and ${\mathbf{C}}_{{{\text{pooled}}}}$ is the pooled covariance matrix between the classes. The QDA classification score for sample $i$ of class $k$ ($Q_{ik}$) is estimated using the variance–covariance for each class $k$ (${\mathbf{C}}_{k}$) in a non-Bayesian form as follows^25,30^:

4 $$Q_{ik} = \left( {{\mathbf{x}}_{i} - { }{\overline{\mathbf{x}}}_{k} } \right)^{{\text{T}}} {\mathbf{C}}_{k}^{ - 1} \left( {{\mathbf{x}}_{i} - { }{\overline{\mathbf{x}}}_{k} } \right)$$

SVM is a binary linear classifier using a nonlinear step that transforms the input sample space into a feature space using a kernel function that maximizes the margins of separation between the sample groups^31^. In this study, a radial basis function (RBF) kernel was used since it is able to adapt to different data distributions^32^. The SVM classification rule is obtained by the following Eq. ^26^:

6 $$f\left( x \right) = {\text{sign}}\left( {\mathop \sum \limits_{i = 1}^{{N_{SV} }} \alpha_{i} y_{i} k\left( {{\varvec{x}}_{i} ,{\varvec{z}}_{j} } \right) + b} \right)$$

where $N_{SV}$ is the number of support vectors; $\alpha_{i}$ is the Lagrange multiplier; $y_{i}$ is the class membership ($\pm$ 1); $k\left( {x_{i} ,z_{j} } \right)$ is the kernel function; and $b$ is the bias parameter. These SVM parameters were obtained and optimized via cross-validation.

### Quality performance

The accuracy, sensitivity, specificity, F-Score and G-Score were calculated in the test set to evaluate the models classification performances. The accuracy represents the total number of samples correctly classified considering true and false negatives, the sensitivity represents the proportion of positive samples (*i.e.*, HIV-infected) correctly classified, the specificity represents the proportion of negative samples (*i.e.*, healthy uninfected controls) correctly classified, the F-score measures the overall classification performance considering imbalanced data, and the G-score measures the overall classification performance not accounting for class sizes^33^. The equations to calculate these parameters are shown in Table 4.

**Table 4:** Equations to calculate the classification quality parameters.

Parameter	Equation
Accuracy (AC) (%)	$\left( {\frac{{{\text{TP}} + {\text{TN}}}}{{{\text{TP}} + {\text{FP}} + {\text{TN}} + {\text{FN}}}}} \right){\text{ x }}100$
Sensitivity (SENS) (%)	$\left( {\frac{{{\text{TP}}}}{{{\text{TP}} + {\text{FN}}}}} \right){\text{x }}100$
Specificity (SPEC) (%)	$\left( {\frac{{{\text{TN}}}}{{{\text{TN}} + {\text{FP}}}}} \right){\text{ x }}100$
F-Score (%)	$\left( {\frac{{2{ } \times {\text{ SENS }} \times {\text{ SPEC}}}}{{{\text{SENS}} + {\text{SPEC}}}}} \right)$
G-Score (%)	$\sqrt {{\text{SENS }} \times {\text{ SPEC}}}$

TP stands for true positive, TN for true negative, FP for false positive and FN for false negative.

## Supplementary information

Supplementary Information.
