# Supplementary material for: ATR-FTIR spectroscopy in blood plasma combined with multivariate analysis to detect HIV infection in pregnant women
Source: Sci Rep. 2020 Nov 19;10:20156. doi: 10.1038/s41598-020-77378-3 (PMC7677535; doi:10.1038/s41598-020-77378-3)
Supplement: Supplementary file 1 — Supplementary Information. [file 41598_2020_77378_MOESM1_ESM.docx]

**Supplementary Material**

**ATR-FTIR spectroscopy in blood plasma combined with multivariate analysis to detect HIV infection in pregnant women**

Lidiane G. Silva^1^, Ana F. S. Péres^2,3^, Daniel L. D. Freitas^1^, Camilo L. M. Morais^4^, Francis L. Martin^5^, Janaina C. O. Crispim^2,3^, Kassio M. G. Lima^1,*^

*^1^Institute of Chemistry, Biological Chemistry and Chemometrics, Federal University of Rio Grande do Norte, Natal 5072-970, RN Brazil*

*^2^Department of Clinical and Toxicological Analysis, Federal University of Rio Grande do Norte, Natal 59072-970, RN Brazil*

*^3^Maternity School Januário Cicco, Federal University of Rio Grande do Norte, Natal 59072-970, RN Brazil*

*^4^School of Pharmacy and Biomedical Sciences, University of Central Lancashire, Preston PR1 2HE, UK*

*^5^Biocel Ltd, Hull HU10 7TS, UK*


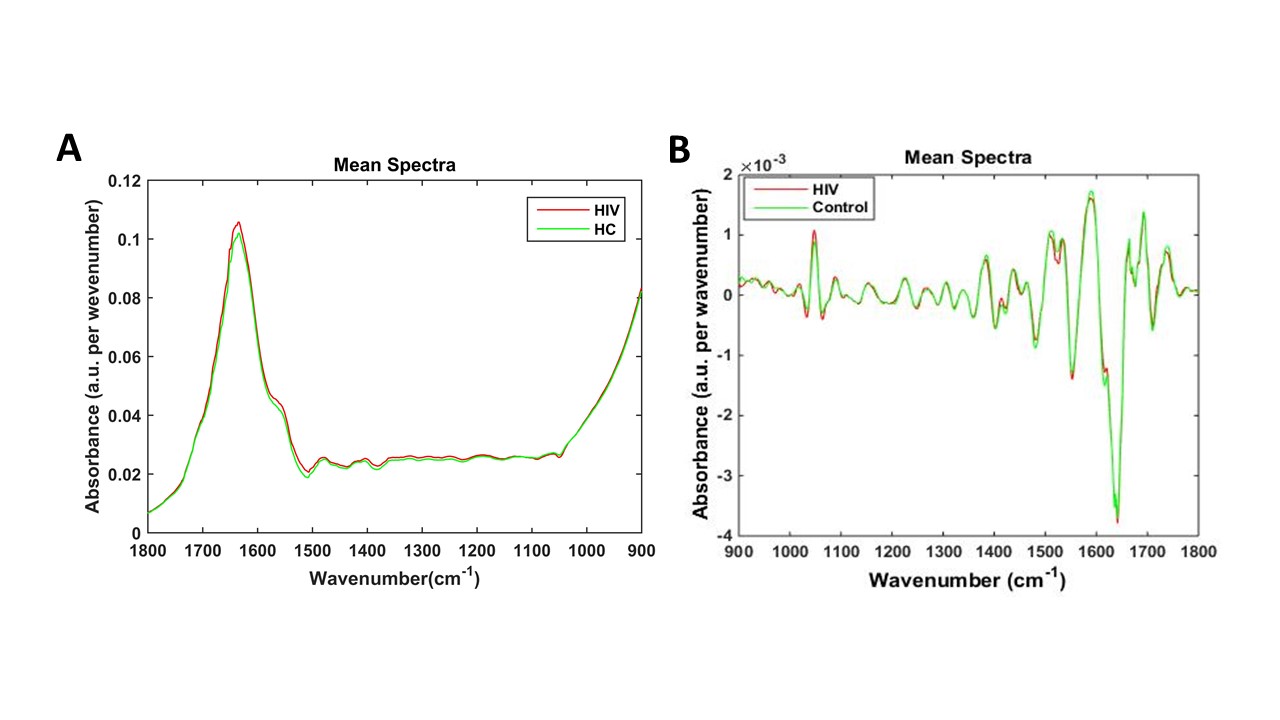


**Figure S1:** **Infrared (IR) spectra.** (A) Average raw IR spectra in the bio-fingerprint region (1800-900 cm^-1^) for samples infected with HIV (HIV) and healthy uninfected controls (HC). (B) Average pre-processed IR spectrum (2^nd^ derivative and normalization) in the bio-fingerprint region (1800-900 cm^-1^) for samples infected with HIV (HIV) and healthy uninfected controls (Control).


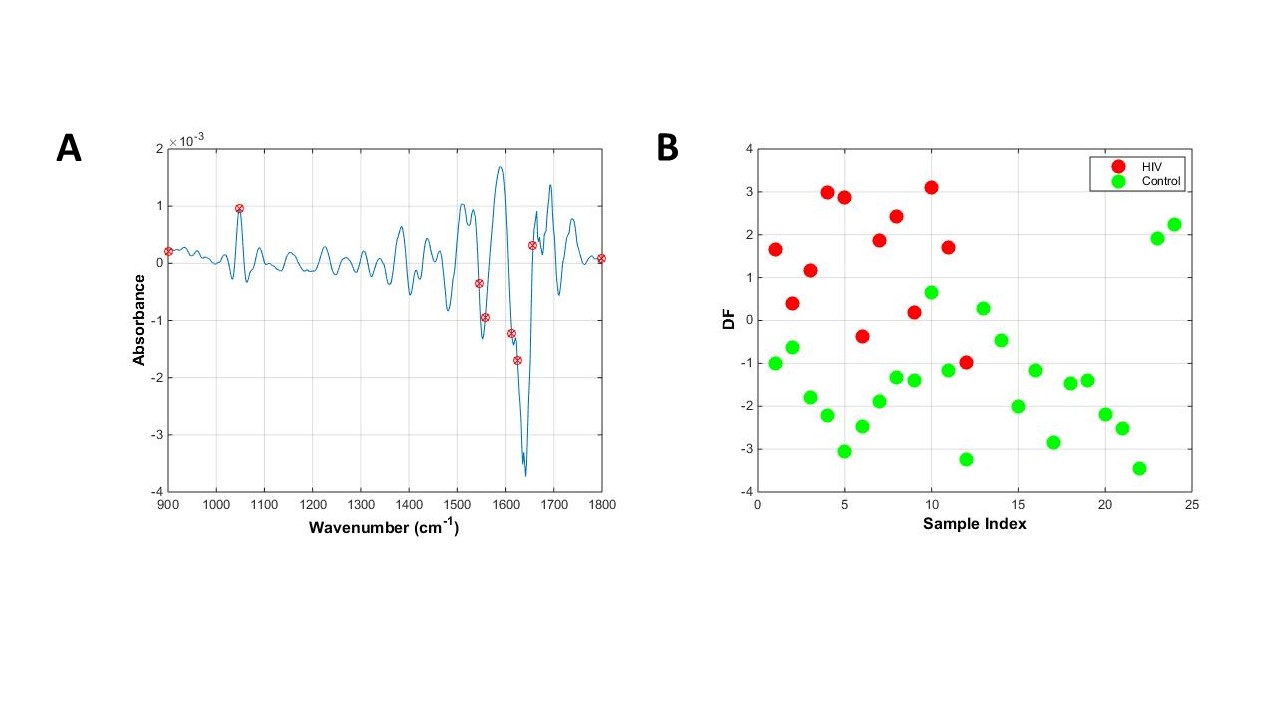


**Figure S2: GA-LDA results.** (A) Selected wavenumbers (901, 1047, 1545, 1558, 1612. 1624, 1657, 1799 cm^-1^) responsible for class separation. (B) Discriminant function (DF) for the samples in the test set, where HIV stands for HIV-infected samples and Control for healthy uninfected controls.

**Table S1:** **Quality parameters calculated in the test set to classify healthy uninfected controls *vs.* HIV-infected samples for genetic algorithm linear discriminant analysis (GA-LDA).** AC = accuracy, SENS = sensitivity, SPEC = specificity

| **Model** | **AC** | **SENS** | **SPEC** | **F-Score** | **G-Score** |
| --- | --- | --- | --- | --- | --- |
| GA-LDA | 83% | 83% | 83% | 83% | 83% |
